# ALDH1 and SALL4 Expression in Cell Block Samples from Patients with Lung Adenocarcinoma and Malignant Pleural Effusion

**DOI:** 10.3390/diagnostics11081463

**Published:** 2021-08-12

**Authors:** Tomohiro Kanayama, Toshiaki Taniguchi, Hiroyuki Tomita, Ayumi Niwa, Kei Noguchi, Mikiko Matsuo, Yuko Imaizumi, Takahiro Kuroda, Yuichiro Hatano, Isao Okazaki, Tatsuo Kato, Akira Hara

**Affiliations:** 1 Department of Tumor Pathology, Gifu University Graduate School of Medicine, 1-1 Yanagido, Gifu 501-1194, Japan; t_knym@gifu-u.ac.jp (T.K.); taniguti@gifu-u.ac.jp (T.T.); v2111026@edu.gifu-u.ac.jp (A.N.); y2111023@edu.gifu-u.ac.jp (M.M.); y2111003@edu.gifu-u.ac.jp (Y.I.); z2111020@edu.gifu-u.ac.jp (T.K.); yuha@gifu-u.ac.jp (Y.H.); ahara@gifu-u.ac.jp (A.H.); 2 Division of Pathology, Gifu University Hospital, 1-1 Yanagido, Gifu 501-1194, Japan; kei_nogu@gifu-u.ac.jp; 3 National Hospital Organization Nagara Medical Center, Department of Clinical Laboratory, Gifu 502-8558, Japan; sakakura.takeshi.sn@mail.hosp.go.jp; 4 National Hospital Organization Nagara Medical Center, Department of Respiratory Medicine, Gifu 502-8558, Japan; kato.tatsuo.ue@mail.hosp.go.jp

**Keywords:** malignant pleural effusion, cancer stem cell, cell block, ALDH1, SALL4

## Abstract

Malignant pleural effusion (MPE) can accompany advanced lung adenocarcinoma. Recent studies suggest that MPE could contain a heterogeneous subpopulation of cells with stem-like properties, such as tumorigenicity and self-renewal, indicating that they could be the source of metastasis. Although previous studies analyzed the correlation between cancer stem cell (CSC) marker expression and clinical outcomes using lung cancer tissues, investigations regarding the association of MPE with CSC marker expression are limited. We performed immunohistochemistry to examine the expression of aldehyde dehydrogenase 1 (ALDH1) and Sal-like 4 (SALL4) in 46 cell block samples of MPE from patients with lung adenocarcinoma. ALDH1-positive and SALL4-positive cancer cells in MPE were detected in 30 (65.2%) and 21 samples (45.7%), respectively. Cluster formation was detected in 26 samples (56.5%). The number of clusters was significantly higher in ALDH1-positive/SALL4-negative samples. SALL4 expression was inversely correlated with the cluster ratio (*r* = −0.356) and positively associated with the Ki-67 index (*r* = 0.326), suggesting that MPE cells with high SALL4 expression comprised the proliferative subpopulation. In conclusion, we demonstrated that MPE contains an ALDH1-positive/SALL4-negative subpopulation exhibiting cluster formation and a SALL4-positive proliferative subpopulation.

## 1. Introduction

Lung adenocarcinoma is the leading cause of cancer mortality globally. Currently available therapies are relatively ineffective, and the age-standardized 5-year survival rate is 10–20% in most countries [1,2]. Treatment failure in patients with lung cancer can be attributed to the presence of a subpopulation of cancer cells with distinctive features, defined as cancer stem cells (CSCs) or cells with stem cell-like properties [3,4,5,6].

As observed in other cancers, CSCs in lung cancer have the ability to generate new tumors by exploiting their stem cell nature, including the ability to self-renew and differentiate into multiple cell lineages [7]. Several specific markers have proven useful for the isolation of subsets enriched for CSCs in lung cancer, including CD133, CD44, and aldehyde dehydrogenase 1 (ALDH1) activity [8].

ALDH1 is an isoform of the ALDH family of cytosolic enzymes responsible for oxidizing intracellular aldehydes [9]. Increased ALDH1 activity has been found in stem cell populations in hematologic malignancies and solid tumors [9,10]. Histological analysis of ALDH1 expression using lung tissue samples revealed a correlation between its expression and poor prognosis in patients with lung cancer [11,12,13]. Thus, inhibition of ALDH activity is expected to effectively eradicate the drug-tolerant CSC subpopulation during lung cancer treatment [14].

Several molecules such as Sal-like 4 (SALL4) and octamer-binding transcription factor 4 (OCT4) have been identified as important factors in maintaining self-renewal and proliferation in stem cells [15,16,17]. SALL4 is a mammalian homolog of the *Drosophila* region-specific homeotic gene spalt (sal), which encodes a multiple zinc finger transcription factor. SALL4 is a chief regulator of transcriptional networks that plays important roles in maintaining stemness in the early stage of development [16,17]. SALL4 upregulation has also been reported, and SALL4 mRNA expression is considered a drug resistance factor in lung adenocarcinoma [18,19]. These results support the hypothesis that SALL4-expressing cancer cells feature cancer stem-like properties, such as tumorigenicity and resistance to therapy.

Malignant pleural effusion (MPE) is defined as pleural fluid containing malignant cells [20]. MPE is a frequent complication of advanced lung cancer, occurring in approximately 30% of patients, and it is associated with poor prognoses [19,20,21]. Further, MPE offers a unique opportunity to culture a wide variety of cancer cells, and this heterogeneity can facilitate the development of subpopulations with cancer stem-like properties [22,23]. The study of MPE could help clarify the mechanism by which cancer cells with stem cell-like properties in effusions contribute to the progression from individual cells to enriched cell clusters in advanced metastatic lung cancer.

In clinical practice, cytology of MPE is a common and important procedure because of its pivotal role in the diagnosis, treatment, and follow-up of patients. In the cell block technique, cytological material is solidified into a pellet and embedded in paraffin blocks for further histological processing. Compared with cytological smears, cell block samples have the advantages of morphological observation and immunohistochemistry (IHC) of tumor cells, and the preparation of serial sections enables pathologists to identify detailed morphological and immunohistochemical features [1,24]. These characteristics of cell blocks are assumed to be helpful for the analysis of tumor cells in malignant effusion. However, research on the utilization of cell blocks has focused mainly on practical uses, such as the evaluation of diagnostic markers or predictive biomarkers, and studies concerning tumor cell properties in cell block samples are limited.

In this study, we analyzed the characteristics of lung cancer cells with stem-like properties using cell block samples prepared from MPE.

## 2. Results

### 2.1. Patient Characteristics

A consecutive series of patients who had MPE and underwent thoracentesis at Nagara Medical Center (Gifu, Japan) between 2012 and 2016 were selected to analyze MPE. All the patients were classified as stage Ⅳ and were untreated at the time of the thoracentesis. Among 78 cases in total, 46 patients were pathologically diagnosed with primary lung adenocarcinoma, and their cell block samples featured MPE. The patients included 27 men (58.7%) and 19 women (41.3%), with a median age of 70.5 years (range, 43–93).

### 2.2. Expression of ALDH1 and SALL4 in Lung MPE Samples

**Figure 1:**
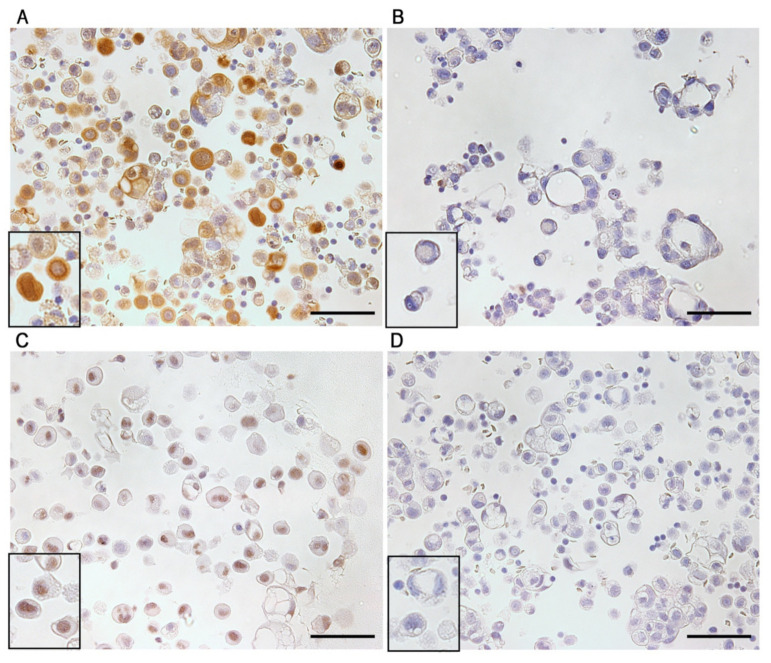
Immunohistochemistry of aldehyde dehydrogenase 1 (ALDH1) and Sal-like 4 (SALL4) expression in the malignant pleural effusion of lung adenocarcinoma. ALDH1-positive (**A**), ALDH1-negative (**B**), SALL4-positive (**C**), and SALL4-negative (**D**) cells. The insets present a higher-magnification image in each panel. The bars indicate 50 µm.

To investigate the expression of ALDH1 and SALL4 in MPE from patients with lung adenocarcinoma, we performed IHC using SALL4 and ALDH1 antibodies and patient-derived MPE cell blocks (Figure 1). ALDH1 expression was predominantly observed in the cytoplasm (Figure 1A). We divided cells into ALDH1-positive and ALDH1-negative subsets according to the findings (Figure 1B). SALL4 expression was detected in the nucleus (Figure 1C), and we divided cells into SALL4-positive and SALL4-negative subsets based on the findings (Figure 1D).

**Figure 2:**
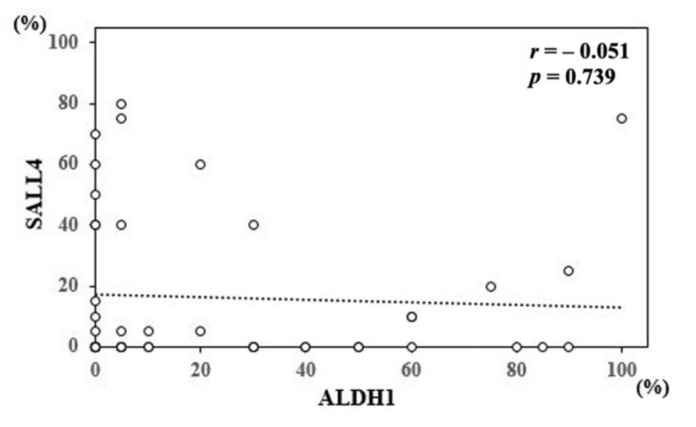
Correlation between aldehyde dehydrogenase 1 (**ALDH1**) and Sal-like 4 (**SALL4**) expression. The expression of ALDH1 was not significantly correlated with that of SALL4.

Consequently, 30 (65.2%) and 21 samples (45.7%) were positive for ALDH1 and SALL4, respectively (Table 1). Seventeen samples (40.0%) were positive for both ALDH1 and SALL4. We analyzed the correlation between ALDH1 and SALL4 expression; however, no significant correlation was noted (Figure 2). These results suggest that ALDH1 and SALL4 expression is, in part, associated with MPE in human lung adenocarcinoma.

### 2.3. Assessment of Cluster Formation in MPE

**Figure 3:**
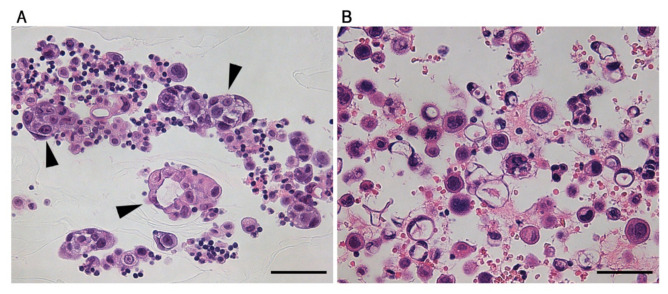
Morphological characterization of malignant pleural effusion (MPE). (**A**) A representative hematoxylin and eosin (HE)-stained image of an MPE with many clusters. Arrowheads indicate clusters. A cluster was defined as a cell assembly consisting of at least five cancer cells. (**B**) A representative HE image of an MPE consisting of almost entirely isolated cells. The bars indicate 50 µm.

It is considered that cancer cells with stem cell-like properties in MPE contribute to the progression from individual cells to enriched cell clusters and advanced metastatic lung cancer [25]. To determine if cluster formation is associated with lung MPE, we assessed cluster formation in our cohort. In this study, we defined a cell assembly that consisted of at least five cells as a cluster (Figure 3A,B). The cluster-rich group included samples with cluster ratios of at least 50%, whereas the cluster-poor group consisted of samples with cluster ratios of less than 50%. The cutoff value of 50% was determined based on a previous report [26]. In the analysis, 27 patients (58.7%) were categorized into the cluster-rich group.

### 2.4. Cluster Formation and Expression of ALDH1 and SALL4 in MPE

**Figure 4:**
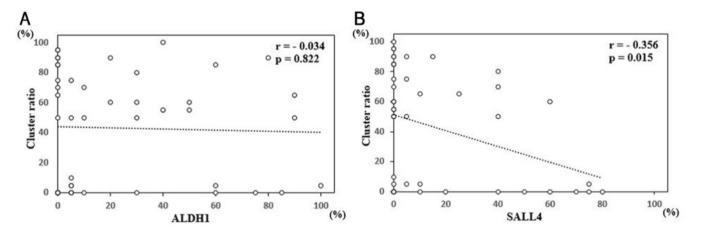
Sal-like 4 (SALL4) expression is inversely associated with cluster formation. (**A**,**B**) Correlation of aldehyde dehydrogenase 1 (ALDH1) (**A**) and SALL4 expression (**B**) with the cluster ratio in patients with malignant pleural effusion.

To elucidate the relationships of cluster formation with ALDH1 and SALL4 expression, we analyzed ALDH1 and SALL4 expression and the cluster number/ratio in our cohort. Samples with an ALDH1-positive/SALL4-negative pattern (17 samples) exhibited significantly more clusters than other groups (*p* < 0.01, Table 2). Next, we tested the correlation between ALDH1/SALL4 expression and the cluster ratio. The cluster ratios in the ALDH1-positive and SALL4-positive groups were 39.2% and 33.8%, respectively. ALDH1 positivity was not correlated with the cluster ratio (*r* = −0.034, *p* = 0.822, Figure 4A), whereas SALL4 positivity was negatively correlated with the cluster ratio (*r* = −0.356, *p* = 0.015, Figure 4B). These results suggest that the subpopulation of ALDH1-positive/SALL4-negative cancer cells in MPE may have a greater capacity for cluster formation.

### 2.5. Increased SALL4-positive Cell Count Is Correlated with Increased Number of Proliferative Cells in MPE

**Figure 5:**
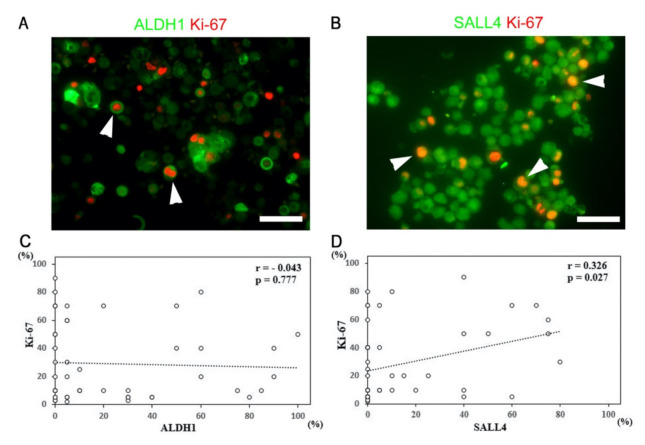
Sal-like 4 (SALL4) expression is positively correlated with cellular proliferation. (**A**) Double immunofluorescence of aldehyde dehydrogenase 1 (ALDH1) and Ki-67 in a patient with malignant pleural effusion (MPE) and high ALDH1 expression. Some cancer cells co-expressed ALDH1 and Ki-67, but Ki-67-positive cells were detected in both ALDH1-positive and ALDH1-negative subpopulations. (**B**) Double immunofluorescence of SALL4 and Ki-67 in a patient with MPE and high SALL4 expression. Ki-67-positive tumor cells were present in the SALL4-positive subpopulation. (**C**,**D**) Analysis of the correlation of ALDH1 or SALL4 expression with Ki-67 expression in cell blocks. SALL4 expression and the Ki-67 index were positively correlated. Arrowheads indicate double-positive cells. The bars indicate 50 µm.

To investigate the correlation of ALDH1 or SALL4 expression with cellular proliferation, we performed double immunofluorescence staining for ALDH1 or SALL4 and Ki-67 antibodies. The results revealed clear ALDH1-positive/Ki-67-positive and SALL4-positive/Ki-67-positive cell populations (Figure 5A,B). Next, we conducted correlation analysis of ALDH1 and SALL4 expression with Ki-67 immunostaining in cell blocks of MPE (Figure 5C,D). SALL4 expression was positively correlated with the Ki-67 index (*r* = 0.326, *p* = 0.027), whereas no relationship was identified between ALDH1 expression and the Ki-67 index (*r* = −0.051, *p* = 0.739).

## 3. Discussion

In this study, we demonstrated the characteristics of lung cancer cells with stem-like properties in MPE using cell block samples prepared from MPE. MPE in advanced lung adenocarcinoma has received increasing attention because of its links to poor prognoses through several roles, such as the maintenance of tumor heterogeneity, retention of CSCs, and promotion of metastasis [25]. However, few reports have assessed the existence of CSCs in effusions, and less is understood about the association between the existence and properties of CSCs and the tumor microenvironment in MPE. Thus, we confirmed the expression patterns of ALDH1 and SALL4 in MPE using cell block samples.

The cell block technique is a valid and useful tool for studying tumor cells in effusions. Collected cells are formalin-fixed and then embedded in paraffin, allowing samples to be handled in the same manner as biopsy or surgical specimens. The efficacy of IHC of cell block samples was previously reported mainly for diagnostic purposes and predictive biomarker testing [1,27]. In this study, we evaluated the morphological properties of tumor cells using hematoxylin and eosin (HE) staining, and the expression of CSC markers was examined using IHC. Moreover, dual immunofluorescence for SALL4 and Ki-67 revealed their correlation.

Our results revealed the presence of ADLH1-positive and/or SALL4-positive cells in most MPE samples. ALDH1 is a well-known CSC marker in many cancers including lung cancer [9,28]. ALDH1 expression has been reported in some lung cancer cell lines, and ALDH1 upregulation could contribute to the malignant transformation of lung cells [12]. Whereas there have been increasing reports of ALDH1-positive lung CSCs, the expression rate of ALDH1 in lung cancer tissue considerably varies among patients [29]. The present study revealed the existence of an ALDH1-positive/SALL-negative population with greater capacity for spheroid formation, a stem-like property. However, the expression of ALDH1 demonstrated by immunohistochemistry in the present study was limited to the ALDH1A1 isoform. Further investigation using fresh or frozen samples is needed to clarify enzymatic activity and isoform specificity in MPE tumor cells.

SALL4 is known to have two types of isoforms—namely, SALL4A and SALL4B. A previous report demonstrated that both isoforms can interact with Nanog and that SALL4B alone could maintain the pluripotent state in murine embryonic stem cells [30]. SALL4 expression in carcinoma cells was recently reported, and SALL4 was suggested to be associated with cell proliferation and epithelial–mesenchymal transition (EMT). Our result affirms previous findings regarding the association between SALL4 overexpression and cell proliferation using SBC-1 lung cancer cells [31].

The role of SALL4 in cell dispersion has been investigated using cell lines of breast cancer [32,33], endometrial cancer [34], and gastric cancer [35]. In breast cancer cells, SALL4 expression is inversely correlated with that of the EMT marker ZEB1, as well as CDH1. In addition, SALL4 knockdown downregulates the expression of a multifunctional transmembrane glycoprotein CD44 in gastric cancer and lung adenocarcinoma cells [35,36]. Accumulating evidence demonstrates that CD44 suppresses cell adhesion and promotes tumor invasion and metastasis in various cancer types [37]. Our finding that SALL4 expression was inversely correlated with cluster formation may have resulted from its role in cell dispersion via upregulation of EMT factors and CD44.

A previous study examined the morphological characteristics of malignant effusion by analyzing the correlation between survival rates and the size of cancer cell clusters. In total, 289 cases of malignant pleuroperitoneal effusion with carcinoma of various origins were reviewed, and the results demonstrated that the larger cluster group (more than 100 cancer cells) had the best prognosis, whereas the group without cluster formation had the worst prognosis [38]. Another report concerning breast cancer revealed that patients with spheroid-rich MPE had a worse prognosis than those with fewer spheroids in MPE [26]. Although those studies supported the correlation between cluster formation of malignant effusion and life expectancy, the association of morphological features with the prognosis of MPE in lung cancer has not been examined. Our analysis of ALDH1 and SALL4 expression suggested that cancer cells in clusters and single cells have different properties of tumorigenicity, but the clinical significance of cluster formation in MPE is unclear. Further research is needed to clarify the relationship between cluster formation and clinical outcomes.

**Figure 6:**
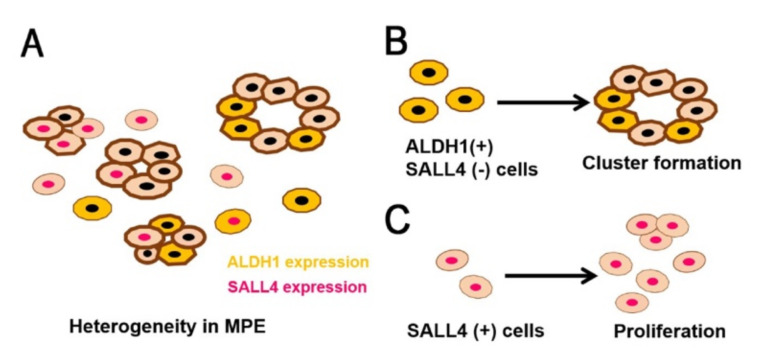
Schematic model of lung adenocarcinoma with malignant pleural effusion (MPE). (**A**) Tumor cells in MPE are heterogeneous. MPE samples exhibited clusters and isolated cells, and some tumor cells expressed aldehyde dehydrogenase 1 (ALDH1) or Sal-like 4 (SALL4). (**B**) The subpopulation consisting of ALDH1-positive/SALL4-negative cells has capacity to form clusters, which is a stem-like property. (**C**) SALL4 expression is related to cellular proliferation.

In this study, we confirmed the expression patterns of ALDH1 and SALL4 in MPE using cell block samples. The hypothesized characteristics of lung adenocarcinoma MPE is presented in Figure 6 as a schematic model. ALDH1 expression was not associated with the cluster ratio, but cluster formation was significantly increased in ALDH1-positive/SALL4-negative samples. A subpopulation with positive ALDH1 and/or SALL4 expression in lung adenocarcinoma MPE could influence tumorigenicity and proliferation, and it might be associated with poor prognosis.

This study is insufficient in terms of the number of patients, clinical information, such as chemotherapy, therapeutic efficacy, detailed histopathological details information of the tumor, and prognosis, so the further accumulation of cases and detailed analysis of clinical data will be necessary. Further, the results of this study do not clarify how the stem cell and clusterization characteristics of the MPE in lung cancer are related to properties of clinical features and prognosis. However, the study on the use of cell blocks is likely to be useful for practical applications, mainly in evaluating diagnostic and predictive biomarkers.

## 4. Materials and Methods

### 4.1. Patients and Samples

In total, formalin-fixed, paraffin-embedded cell block samples were collected from patients who had MPE and who underwent thoracentesis at Nagara Medical Center between 2012 and 2016. All specimens were received as fresh unfixed effusions (volume range, 20–2000 ml). The cell block samples were prepared using glucomannan, as described previously [39]. The specimens were centrifuged, and each resulting pellet was fixed with formalin. The fixed effusion was stained with eosin solution and suspended in 80% alcohol. Then, glucomannan–formalin water solution (Holdgel 110; Asiz Kizai, Tokyo, Japan) was added. After immersion in methanol for 2 h, glucomannan solidified and became gelatinous. Then, each sample was dehydrated and infiltrated with paraffin, and a paraffin-embedded block was prepared.

Sections (3 µm) of all cell block samples were stained with HE. These slides were observed to confirm the diagnosis of lung adenocarcinoma. Patients diagnosed with lung squamous cell carcinoma, metastatic adenocarcinoma, or malignant mesothelioma were excluded.

Moreover, cluster formation was assessed using HE-stained slides in a double-blinded manner by two pathologists (T.K. and H.T.). A cell assembly consisting of at least five tumor cells was defined as a cluster. The number of clusters in five low-power fields (×100 magnification) was counted in each case. The cluster ratio was calculated as the proportion of tumor cells in a low-power field that were included in clusters. The serial 3 µm thick sections were used for IHC and immunofluorescence.

All parts of this study were approved by the Institutional Review Board of Gifu University Hospital and Nagara Medical Center.

### 4.2. IHC

Paraffin-embedded cell block samples of MPE were used for IHC. These tissues were cut into 3 µm thick sections. They were subjected to heat pretreatment at 65 °C for 40 min. Slides were deparaffinized in xylene and rehydrated through a graded alcohol series. Sections were then heated at 120 °C for 1 min in 10 mM citrate buffer for antigen retrieval using Pascal. After the slides were immersed in 0.3% H_2_O_2_ in methanol to quench endogenous peroxidase activity, they were blocked with 2% bovine serum albumin. Each section was incubated with primary antibody at 4 °C overnight. Then, the slides were incubated with secondary antibody conjugated to VECTASTAIN Elite ABC kit (Funakoshi, Japan) at room temperature for 30 min and amplified with VECTASTAIN Elite ABC kit at room temperature for 30 min. The reaction products were visualized using ImmPACT DAB. After counterstaining with hematoxylin, each slide was sealed.

To investigate human ALDH1 expression, anti-ALDH1 (ALDH1A1 isoform, clone 44, human ALDH1 aa 7-128, mouse monoclonal IgG, 1:100, BD Transduction Laboratories) was used as the primary antibody. Anti-SALL4 (mouse polyclonal IgG, 1:100, BIOCARE) and anti–Ki-67 (rabbit polyclonal IgG, 1:100, abcam) were also used as primary antibodies to evaluate formalin-fixed, paraffin-embedded cell block samples.

### 4.3. Immunofluorescence

For immunofluorescence, the procedures from pretreatment to incubation with primary antibody followed those of IHC. The slides were incubated with secondary antibody (anti-rabbit IgG, 1:200, abcam; anti-mouse IgG, 1:200, abcam) in the dark at room temperature for 30 min. After incubation with DAPI solution (1:1000, WAKO) in the dark at room temperature for 5 min, each slide was sealed.

### 4.4. Statistical Analysis

Data were tabulated using Microsoft Excel 2007 and analyzed using StatMate V software (Atoms, Tokyo, Japan). The association of ALDH1 and SALL4 expression patterns with the average cluster numbers was analyzed using Fisher’s exact test. The correlations between ALDH1 and SALL4 expression and between Ki-67 and ALDH1 or SALL4 expression were analyzed using a linear regression test.

## 5. Conclusions

We demonstrated the expression patterns of ALDH1 and SALL4 in MPE of lung adenocarcinoma. Cluster formation was significantly increased in ALDH1-positive/SALL4-negative samples, suggesting that such tumor cells contain a subpopulation with a greater capacity for cluster formation. SALL4 expression was inversely correlated with the cluster ratio and was positively associated with high Ki-67 expression. Therefore, the SALL4-positive cell subpopulation in lung adenocarcinoma MPE influences cancer cell proliferation.

## Figures and Tables

**Table 1:** Comparative analysis of cells positive and negative for ALDH1 and SALL4 in patients with MPE.

	Case Number (%) *N* = 46	
	Positive	Negative
**ALDH1**	30 (65.2%)	16 (34.8%)
**SALL4**	21 (45.7%)	25 (54.3%)

**Table 2:** Comparative analysis of the cluster number and ALDH1 and/or SALL4 positive and negative cells in patients with MPE.

	ALDH1-Positive	ALDH1-Negative
**SALL4-Positive**	30.46 (*n* = 13)	50.63 (*n* = 8)
**SALL4-Negative**	103.41 (*n* = 17)	57.13 (*n* = 8)

*p* = 0.00007. The cluster number in each subpopulation was counted in five low-power fields (100-fold magnification). *p*-Value was tested by Fisher’s exact test.
